# Artérias Coronárias Após a Operação de Jatene para Transposição das Grandes Artérias: O Papel da Angiografia Coronária por Tomografia Computadorizada no Seguimento

**DOI:** 10.36660/abc.20210403

**Published:** 2021-06-08

**Authors:** Antonio Joaquim Marinho-da-Silva

**Affiliations:** 1 Centro Hospitalar Universitário de Coimbra Coimbra Portugal Centro Hospitalar e Universitário de Coimbra , Coimbra - Portugal

**Keywords:** Cardiopatias Congênitas/cirurgia, Operação das Grandes Artérias, Comunicação Interventricular, Estenose Aórtica Subvalvar, Circulação Coronária

A correção da transposição das grandes artérias (TGA) pela técnica de Jatene foi uma das maiores conquistas da cirurgia cardíaca nas cardiopatias congênitas. ^1^ Ao substituir as artérias transpostas em seu arranjo anatômico adequado, essa técnica — cirurgia de troca atrial (CTV) — atinge uma relação arterial espacial normal. No entanto, esse novo arranjo requer a excisão dos botões da artéria coronária e seu implante na nova aorta. A dificuldade dessa etapa está relacionada ao tipo de origem coronariana e à distância ao novo local de inserção.

Uma vez concluída essa etapa de normalização anatômica, busca-se a normalização funcional cardíaca. As complicações pós-cirúrgicas mais comuns estão relacionadas à complexidade das anomalias associadas (comunicação interventricular, coarctação da aorta, estenose valvar e outras), locais de sutura dos neovasos, ramificação pulmonar e dilatação da neoaorta. ^2^ Não negligenciáveis e preocupantes, as complicações tardias da circulação coronariana congênita ou adquirida são bem conhecidas. ^3^ A incidência real dessas complicações varia de 0,8% a 27,5%, de acordo com a série de relatos e o tempo de seguimento. ^3 , 4^

O mecanismo das complicações coronarianas é bem conhecido e descrito, variando desde distorção anatômica, ângulo agudo, *kinking* , curso interarterial coronariano, até diferentes tipos de estenose, principalmente estenose ostial crítica, que pode ser fatal. ^3 , 5^ O melhor método para avaliação geométrica ou funcional da circulação coronária ainda é objeto de pesquisa e discussão. Nesta edição dos ABC, o artigo de Baldo et al., ^6^ aborda essa importante questão. Embora funcionalmente assintomáticos, 3,3% desses pacientes demonstraram alterações coronárias possivelmente significativas. O estudo foi de recorte basal, independente dos sintomas. Embora algumas diretrizes internacionais apoiem essa visão ^7^ outras não. ^8^

A maioria dos problemas e eventos coronários descritos até agora tendem a ocorrer na infância, nos primeiros anos após a cirurgia, onde as queixas são de difícil acesso. Além disso, devido à falta de sensibilidade do exame, a abordagem de triagem convencional por eletrocardiograma ou ecocardiografia Doppler nem sempre é útil. Portanto, parece lógico investigar pacientes após a CTV em busca de alterações nas artérias coronárias, apesar da aparente “normalidade”. Antigamente, fazíamos esse rastreamento usando angiografia convencional com exposição desnecessária à radiação e complicações oriundas do cateterismo.

Desde o início do ano 2000, a angiografia coronária por tomografia computadorizada tem assumido um papel cada vez mais importante na avaliação das anomalias coronárias, particularmente após a correção da TGA pela técnica de Jatene. ^9^ Identificar as anomalias em pacientes que podem, em última instância, comprometer suas vidas é o desafio que esse texto apresenta, reforçando a importância de um método de diagnóstico por imagem. Devido às lesões ostiais, a angiografia coronária convencional pode não as identificar. Concordo que pelo menos uma avaliação basal da circulação coronária em todos os pacientes, no pós-operatório, seria razoável. Então, deve-se destacar a superioridade diagnóstica da angio-TC em relação à angiografia coronariana convencional. ^10^ A angiografia por ressonância magnética (RMC) cardíaca e coronariana (evitando a radiação) também pode ser uma opção para avaliar a patência coronariana. ^11^ Uma nova técnica de TC, como a TC de dupla energia combinando perfusão com visualização anatômica, poderá ser mais útil em alguns casos particulares também. ^12^ Será necessário seguimento anual multimodal com ecocardiografia com Doppler, ecocardiografia e tomografia computadorizada ou teste ergométrico de esforço, conforme enfatizam os autores. Mas outras técnicas não invasivas como a análise de deformação derivada do Doppler e taxa de deformação ( *strain rate* ) podem ser úteis na avaliação funcional em vez do ultrassom convencional. ^13^ Obviamente, se forem encontrados sintomas clínicos (arritmia, dor torácica ou fadiga excessiva) ou alterações nos testes padrão, a avaliação cardíaca por tomografia computadorizada coronariana (ou RMC) deve ser feita imediatamente a qualquer momento.

Mas em que idade a avaliação coronária basal na CTV ainda permanece em discussão. Apesar da ausência de sintomas, na minha opinião, deve ser antes da idade adulta. E é prudente fazer tal avaliação também em adultos, sobre os quais ainda não se tem essas informações . ^14^
