# Supplementary material for: Artérias Coronárias Após a Operação de Jatene para Transposição das Grandes Artérias: O Papel da Angiografia Coronária por Tomografia Computadorizada no Seguimento
Source: Arq Bras Cardiol. 2021 Jun 8;116(6):1117–8. [Article in Portuguese] doi: 10.36660/abc.20210403 (PMC8288544; doi:10.36660/abc.20210403)
Supplement: Translation

# Translation: Coronary Arteries after Jatene Operation for Transposition of Great Arteries: The Role of CT Coronary Angiography on Follow-up

**Authors:** Antonio Joaquim Marinho-da-Silva

**Affiliations:** 1 Portugal Centro Hospitalar e Universitário de Coimbra, Coimbra- Portugal

**Keywords:** Heart Defects Congenital/surgery, Arterial Switch Operation, Heart Septal Defects, Ventricular, Aortic Stenosis, Subvalvular, Coronary Circulation

The correction of transposition of the great arteries (TGA) by the Jatene technique was one of the greatest achievements of cardiac surgery in congenital heart disease.^1^ By replacing the transposed arteries in their proper anatomical arrangement, this technique — atrial switch operation (ASO) – achieves a normal spatial arterial relationship. However, this new arrangement requires excision of the coronary artery buttons and their implantation in the new aorta. The difficulty of this step relates to the type of coronary origin and distance to the new insertion site.

Once this anatomical normalization step has been achieved, cardiac functional normalization is sought. The most common post-surgical complications are related to the complexity of associated anomalies (ventricular septal defect (VSD), coarctation of the aorta, valve stenosis, and others), suture sites of the neovessels, pulmonary ramification and dilation of the neoaorta,^2^ Not neglectable and object of concern, late complications of either congenital or acquired coronary circulation are well known.^3^ The actual incidence of such complications ranges from 0,8% to 27,5% according to reporting series and time of follow-up.^3 , 4^

The mechanism of coronary complications is well known and described, going from anatomic distortion, acute angle, kinking, coronary interarterial course to different types of stenosis, particularly critical ostial stenosis that may be life-threatening.^3 , 5^ The best method to either geometrical or functional coronary circulation evaluation is still a matter of discovering and discussion. In this issue of ABC, the article by Baldo et al.^6^ addresses this important question. Although functionally asymptomatic, 3,3% of these patients revealed potentially significant coronary abnormalities. This was a basal study, regardless of symptoms. Although some international guidelines support this view,^7^ others do not.^8^

Most coronary problems and events described so far tend to occur in childhood in the first few years after surgery where complaints are difficult to access. Furthermore, owing to lack of testing sensitivity, conventional screening approach by electrocardiogram ECG or Doppler echocardiography are not always helpful. So, it seems logical to investigate patients after ASO looking for coronary artery abnormalities despite apparent “normality”. In the past, we did this screening using conventional angiography with unnecessary exposure to radiation and catheterization complications.

Since the beginning of the year 2000, coronary angiography by computed tomography (CCT-angiography) has assumed an increasingly important role in the assessment of coronary anomalies, namely after TGA correction by the Jatene technique.^9^ Identifying those anomalies in patients that can ultimately compromise their lives is the challenge that this text poses, reinforcing the importance of one method of diagnostic imaging. Owing to ostial lesions, conventional coronary angiography may not eventually identify them. I agree that at least one basal evaluation of coronary circulation in all post-operative patients would be reasonable. Then, diagnostic superiority of CT angiography compared to conventional coronary angiography should be highlighted.^10^ Cardiac and coronary magnetic resonance angiography (CMR) (avoiding radiation) may also be an option for evaluating coronary patency.^11^ Eventually, a new CT technique like dual-energy CT combining perfusion with anatomic visualization will be more useful in some particular cases, too.^12^ Annual multi-modality follow-up with Doppler echocardiography, echocardiography, and CPT or ergometric stress test will be necessary as the authors emphasize. But other non-invasive techniques such as Doppler-derived strain and strain rate may eventually be helpful in functional evaluation rather than conventional ultrasound.^13^ Of course, if clinical symptoms (arrhythmia, thoracic pain or excessive fatigue) or abnormalities are found in the standard tests, cardiac evaluation by coronary computed tomography (or CMR) should be done immediately at any time.

But at what age should basal coronary assessment in ASO be performed still remains in discussion. Despite the absence of symptoms, in my opinion, it should be before adulthood. And it should be prudent to do such assessment also in adults on whom this information has not yet been obtained.^14^
